# Distal Airway Impairment in Obese Normoreactive Women

**DOI:** 10.1155/2013/707856

**Published:** 2013-08-27

**Authors:** Grégory Marin, Anne Sophie Gamez, Nicolas Molinari, Djamila Kacimi, Isabelle Vachier, Fabrice Paganin, Pascal Chanez, Arnaud Bourdin

**Affiliations:** ^1^Department of Respiratory Diseases, Montpellier University Hospital and INSERM U106, Montpellier University, 34090 Montpellier, France; ^2^Department of Medical Information, Montpellier University Hospital, 34090 Montpellier, France; ^3^Department of Respiratory Diseases, Reunion Hospital, BP 350 97448 Saint-Pierre de la Reunion, France; ^4^Department of Respiratory Diseases, Marseille Hospital and Immunology Laboratory INSERM CNRS U600, UMR6212, Aix-Marseille University, 13284 Marseille, France

## Abstract

*Background*. Asthma-like symptoms are frequent in overweight and obesity, but the mechanism is unclear when airway hyperresponsiveness (AHR) is lacking. In this study, we focused on obese women with a clinical suspicion of asthma but negative methacholine challenge and tested distal airway hyperreactivity, explored by Forced Vital Capacity dose-response slope (FVC DRS). *Objective*. To question AHR at the distal airway level in obese women. *Methods*. A total of 293 symptomatic obese and nonobese women free of treatment were investigated. Methacholine challenge tests were undertaken, and patients were divided according to their results to the test. In hyperreactive and nonhyperreactive patients and in our total population, correlations, regression analyses, and analyses of covariance were performed to compare distal airway hyperreactivity in three groups of body mass index (BMI). *Results*. After adjusting for age and baseline respiratory values, the relationship between FVC and FEV1 (forced expiratory volume in one second) DRS was influenced by BMI, with a lower slope in obese than overweight and normal patients in our total population (*P* = 0.008) and in our nonhyperreactive one (*P* = 0.028). *Conclusion*. Distal airway hyperresponsiveness was observed in symptomatic wheezing obese women negative to methacholine challenge.

## 1. Introduction

The relationship between obesity and airway hyperresponsiveness (AHR) has been a great matter of interest among the scientific community for many years. Several studies have enlightened that obese patients (body mass index (BMI) > 30 kg/m²) were more likely to have a clinical diagnosis of asthma and to receive antiasthma drugs, but the effect of obesity on airway hyperresponsiveness remains debated [1–4].

 Overweight and obesity are comorbid conditions known to be associated with poorer asthma control [5] and potential resistance to current therapy [6]. However, other studies have challenged these associations [7], and the potential benefit of weight loss on bronchial reactivity was questioned [8]. 

 Previous reports demonstrated that obesity was an independent risk factor for AHR [9, 10], but the symptomatic mechanisms in patients free of AHR are to be addressed.

 Asthma-like symptoms are frequent but not always related to AHR—with an important risk of bias in epidemiological studies, where challenges are rarely done (creating a risk of overdiagnosis). In a study by Stenius-Aarniala et al. [11], obese patients had more severe symptoms when taking more asthma medications, but there was no evidence of airflow obstruction.

 Previous studies have hypothesized that the variations in airway calibre and pulmonary volumes, occurring during bronchoconstriction, were different in obese and nonobese asthmatic patients, due to a greater involvement of distal airways in the obese population [12, 13]. However, what this involvement means from a physiological point of view is unclear, and no alternative diagnoses are proposed in these symptomatic patients who did not respond to methacholine.

 Airway closure has been found to be a determinant of AHR. Chapman and his team [14] came to this conclusion by comparing airway closure during bronchoconstriction in asthmatic, nonasthmatic, and nonasthmatic obese subjects. Airway closure and airway narrowing, measured by spirometry, suggested a link between airway closure and AHR. Hence it was important to study the association between obesity and airway closure.

The Forced Vital Capacity fall throughout a methacholine challenge test was previously reported as potential reflection of small airway closure in several studies [15–17].

Besides, the role of gender in AHR in overweight and obese people is a potential confounder in many studies. According to epidemiological reports [18], bronchial asthma or AHR has been found to be associated with obesity only in males [19, 20], only in females [21], or in both genders [22]. For this study, we decided to include only women, in order to maintain homogeneity and to avoid any effect due to gender.

 In the present study, we investigated symptomatic obese women aiming at demonstrating any difference at the distal level of the airway tree. We assessed the impact of BMI on several lung function parameters derived from the methacholine challenge, such as FVC fall and FEV1 fall throughout the methacholine challenge test, and FEV1 and FVC dose-response slopes, in order to better understand the complex relationships between obesity, airway closure, AHR, and asthma-like symptoms.

## 2. Methods

### 2.1. Study Design

 The study was based on the retrospective analysis of subjects from the Respiratory Department of the Arnaud de Villeneuve Hospital (Montpellier, France). 

 According to the French Law (Law 88-1138 relative to Biomedical Research of December 20, 1988, modified on August 9, 2004), this noninterventional study did not require approval by an ethics committee or informed signed consent from patients. It was reviewed and approved by our institutional review board (IRB number: 11/10-07).

 A total of 293 consecutive women were considered from January 2007 to December 2009. All patients had symptoms likely to suggest asthma, which include wheezing, dyspnoea, chest tightness, or cough in the following circumstances: (1) with exposure to cold air, (2) after exercise, (3) during respiratory infections, (4) following inhalant exposures in the workplace, and (5) after exposure to allergens and other asthma triggers [23]. Differential diagnoses were excluded according to good clinical practice by the physicians [24], subjects were free from any asthma medication, and smokers greater than 5 packs per year were excluded. 

All patients underwent a methacholine bronchial provocation test, and AHR was defined as a provocative concentration causing a 20% fall in FEV1 (PC20) inferior or equal to 16 mg/mL. Throughout this paper, positive patients to the methacholine test are referred to as “AHR+” and negative patients as “AHR−.”

 Three groups of patients were considered according to their BMI. BMI-normal subjects were defined as having a BMI < 25 kg/m^2^, overweight subjects as having a BMI ≥ 25 kg/m^2^ and < 30 kg/m^2^, and obese subjects as having a BMI ≥ 30 kg/m^2^ [25].

### 2.2. Methacholine Challenge

 Methacholine challenges were performed according to the American Thoracic Society Guidelines [23], with the five-breath dosimeter method (ADD AerodoseR with Atomisor NL11AD nebulizer, Atomisor, CE 0459, Dir. Eur. 93/42/CEE). After the baseline spirometry was performed, the first dose of methacholine was administered if the FEV1 value was greater than 80% of the predicted value. The FEV1 was measured after the appropriate amount of time (about 30 seconds). We made sure to obtain an acceptable-quality FVC at each dose, without too much manoeuvres in order to avoid tiredness. The quality control for FVC repeatability was done by a calibrated machine coupled to a professional software (JAEGER). 

 Furthermore, in order to keep the cumulative effect of methacholine, the waiting time between each dose was kept under 2 minutes. When an FEV1 fall greater or equal to 20% was reached, a bronchodilator was administered and FEV1 was measured after a 15-minute wait and until complete recovery was reached.

AHR was defined by a PC20 lower or equal to 16 mg/mL. 

### 2.3. Variables

 AHR was measured as the FEV1 dose-response slope (DRS) throughout the methacholine challenge test. Distal airway closure was estimated by the FVC DRS. These DRS were obtained from a calculation based on the last value minus the baseline value, divided by the last methacholine dose in mg. With this methodology, we could take into account the methacholine dose when considering the fall in respiratory parameters. The relationship between changes in FVC and FEV1, each represented by their dose-response slope, provided an estimate of the proportion of FEV1 change that was attributable to airway closure.

The closing index was the percentage fall in FVC divided by the percentage fall in FEV1. The percentage falls were defined as (last value − baseline value)/last value.

*Implemented Variables*

(1) FEV1  DRS  =Forced  Expiratory  Volume  Dose   −Response  Slope  =loss  (in  mL)  of  the  maximal  amount     of  air  forcefully  exhaled  in  one  second,   per  mg  of  methacholine  =(Last  FEV1  value−baseline  FEV1  value)Last  methacholine  dose,FVC  DRS  =Forced  Vital  Capacity  Dose−Response  Slope  =loss  (in  mL)  of  vital  capacity  from   a  maximally  forced  expiratory  effort,   per  mg  of  methacholine  =(Last  FVC  value−baseline  FVC  value)Last  methacholine  dose,Percentage  Fall  in  FEV1    =(Last  FEV1  value−Baseline  FEV1  value)Last  FEV1  value,Percentage  Fall  in  FVC  =(Last  FVC  value−Baseline  FVC  value)Last  FVC  value,Closing  Index=(Percentage  Fall  in  FVC)(Percentage  Fall  in  FEV1).

### 2.4. Statistical Analyses

 First, a Kolmogorov-Smirnov test was used to assess the normality of our variables. Because variables were not normally distributed, the comparison of population characteristics between normal weight, overweight, and obese patients was studied via nonparametric Kruskal-Wallis tests, and the results were expressed as medians and interquartile ranges. Nonparametric Spearman correlation tests were also executed, in order to quantify the strength of the relationship between FEV1 and FVC fall.

The effect of BMI on the relationship between the fall in FEV1 and the fall in FVC throughout the methacholine test was examined using a linear regression of FVC DRS against FEV1 DRS for the three levels of BMI (normal, overweight, and obesity), with BMI-normal patients as our control group. FEV1 DRS was considered as the predicted variable, and FVC DRS as the independent parameter. This allowed us to calculate the contribution of the FVC DRS to the FEV1 DRS.

In order to compare the slopes calculated in the linear regressions and represented in the graphic representations, we carried out analyses of covariance (ANCOVA), with FEV1 DRS as the predicted variable and FVC DRS and BMI as the independent parameters. We focused particularly on the interaction term, which described significant differences between the slopes.

This has been done for the AHR+ patients, the AHR− patients, and the whole population. In order to achieve normality and a clearer graphic representation, log (FEV1 DRS) and log (FVC DRS) were used instead of the variables themselves. 

All these results were adjusted for age and baseline respiratory values, and a *P* value lower than 0.05 was considered statistically significant.

Statistical tests were performed with R 2.10 (the R Foundation for Statistical Computing) and SAS 9.2 (SAS Institute Inc., Cary, NC).

## 3. Results

### 3.1. Demographics and Body Mass Index

 Baseline demographics and clinical characteristics categorized according to BMI groups are shown in Tables 1, 2, and 3. 182 women with normal BMI, 62 overweight, and 49 obese participated in this study. Overweight and obese women were older (*P* < 0.0001) and had lower values in baseline FVC and FEV1 (*P* < 0.0001) and FEV1/FVC ratio (*P* = 0.0375). Obese patients also had higher values in FEV1 fall (*P* = 0.0230), FEV1 DRS (*P* = 0.0008), FVC fall (*P* = 0.0011), and FVC DRS (*P* = 0.0008) (Table 1).

AHR+ patients differed only by age and baseline FEV1. In this subgroup, overweight and obese women were significantly older (*P* < 0.0001) and had lower values in baseline FEV1 (*P* < 0.0001) (Table 2). Considering the AHR− population (Table 3), overweight and obese patients were older (*P* = 0.0003) and had lower values in baseline FEV1 and FVC (*P* < 0.0001). 

### 3.2. Airway Closure and Obesity

#### 3.2.1. Correlation between FEV1 DRS and FVC DRS

Whether it be in our whole population, AHR+ patients, or AHR− patients, the relationship between FEV1 DRS and FVC DRS was strong, particularly for the obese patients in the entire population, where the variability of FVC accounted for about 90% of the variability of FEV1 (*R* ^2^ = 0.90), and the Spearman coefficient was 0.97 (Table 4). 

Considering the AHR+ patients, the FVC variability accounted for about 80% of the variability of FEV1, and the Spearman coefficients were between 0.83 (BMI-normal patients) and 0.93 (obese patients).

In the AHR− population, the values were again high, particularly for the obese patients, with an *R* ^2^ of 0.77 and a Spearman coefficient of 0.87.

#### 3.2.2. Impact of BMI on the Relationship between FEV1 DRS and FVC DRS Analyzed by ANCOVAs, after Adjusting for Age and Baseline Respiratory Values

**Figure 1:**
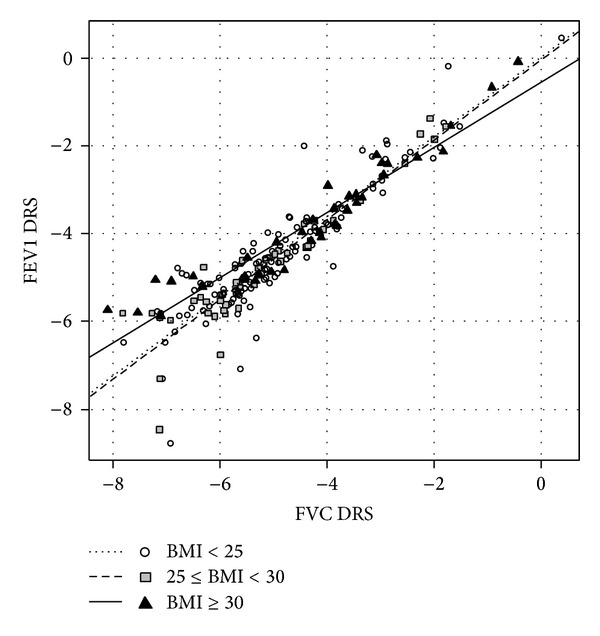
Relationship between FVC dose-response slope and FEV1 dose-response slope, log-transformed, in the entire population, after adjustment for age and baseline respiratory values. Linear regression lines for each level of BMI are shown.

*Entire Population.* The relationship between FVC DRS and FEV1 DRS was significantly different according to BMI, with a lower slope in obese than overweight and normal patients (0.74 (sd: 0.04) versus 0.91 (0.04) and 0.90 (0.05), resp., *P* = 0.008) (Figure 1, Tables 5 and 6).

**Figure 2:**
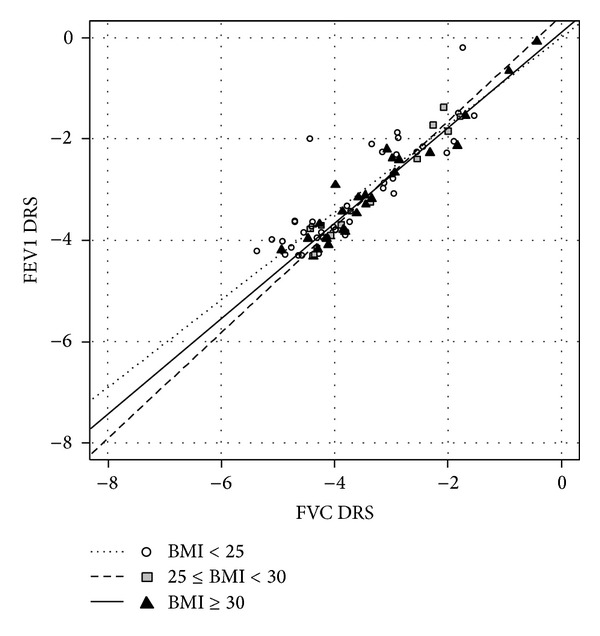
Relationship between FVC dose-response slope and FEV1 dose-response slope, log-transformed, in the AHR+ population, after adjustment for age and baseline respiratory values. Linear regression lines for each level of BMI are shown.

*AHR+ Patients*. No difference was found in the AHR+ population, with slope values of 0.87 (0.06), 1.04 (0.06), and 0.94 (0.06) (*P* = 0.409) for the BMI-normal, the overweight, and the obese patients, respectively (Figure 2, Tables 5 and 6).

**Figure 3:**
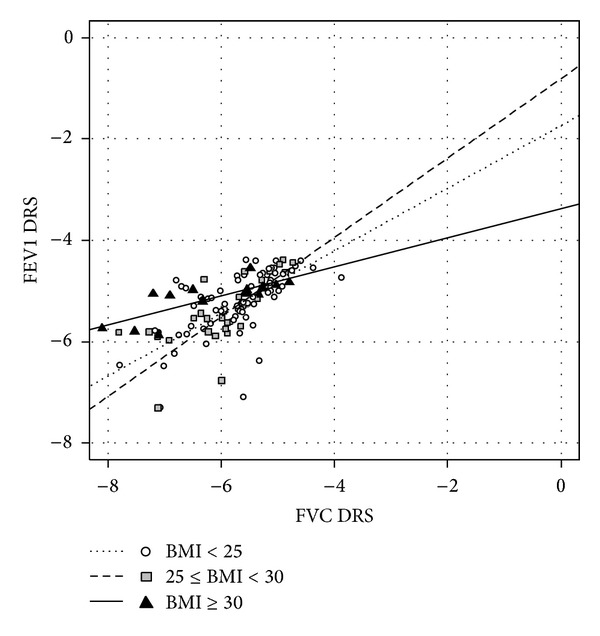
Relationship between FVC dose-response slope and FEV1 dose-response slope, log-transformed, in the AHR− population, after adjustment for age and baseline respiratory values. Linear regression lines for each level of BMI are shown.

*AHR− Patients*. The methacholine-negative patients were found to have very different slopes, with a lower slope in obese than overweight and normal patients (0.29 (0.06) versus 0.78 (0.14) and 0.62 (0.09), resp., *P* = 0.028) (Figure 3, Tables 5 and 6).

## 4. Discussion

### 4.1. Main Results

 In obese women tested for AHR, we observed increased dose-response slope of FVC plotted against FEV1, in the whole population and in AHR− patients. According to the graphic representations, the regressions, and the analyses of covariance, we observed a greater contribution of FVC fall to the FEV1 fall for obese women. 

Our results highlight the importance of obesity on the involvement of distal airways. On the patients with asthma-like symptoms but negative challenge, obesity could increase airway resistance via distal airways [26], thus leading to distal AHR (i.e., FVC fall throughout the methacholine challenge test) but not to proximal one [14]. Indeed, the problem in observing methacholine-positive patients is that focusing on distal airway hyperreactivity is made tricky by the proximal airway hyperreactivity, which “hides” any other part of the airway tree. 

 Increased closing index and classical improvement at exercise of a certain degree of hypoxia at rest are other reflections of small airway involvement [27]. But to the best of our knowledge, this is the first time that distal impairment in symptomatic but not hyper responsive women is dynamically observed and challenged by nonspecific parasympathomimetics. Whether this phenomenon can be reached by any kind of medication remains a critical issue to address as weaning from inhaled corticosteroids, and bronchodilators remained free of life-threatening event [28]. 

### 4.2. Implications for Research and Clinical Relevance

Nitrogen slopes or respiratory CT scan in these patients might be worth investigation to achieve a better understanding of the mechanisms involved in the impact of obesity on distal airways. This could lead to better treatments for wheezing obese patients, who are clearly a complex phenotype. A more specific medication, treating especially distal airways, might be worth investigation in these patients. 

 Unfortunately, symptoms were not quantified, and lung volumes were not prospectively recorded. But rigorousness of the protocol and methacholine dosage nonetheless allowed us to observe an expected difference.

 As in every functional study dedicated to BMI influences, result expression is a critical issue to address, as equations are including the BMI. Changes in percentage of flows and volumes are then highly affected by the pretest values, that is to say that FEV1 fall is easier to observe in the obese with lower starting FEV1 values. Most of our results were clearly influenced by basal values of flows and volumes, and an adjustment was required.

 Some authors [15–17] reported that FVC fall during methacholine test was a good indicator of small airway closure. We decided to compute dose-response slopes because it was applicable to nonhyperreactive patients. This allowed us to analyse these data in this particular subpopulation, where the diagnostics are often tricky [28].

Previous studies already suggested that increased abdominal pressure on the diaphragm in the obese can lead to a stronger contribution of airway closure [29, 30]. Even though obesity decreases baseline pulmonary capacity by altering respiratory physiology [31], the contribution of distal airway closure to the change in FEV1 has been shown to be affected by obesity. However, the mechanical hypothesis—loss of elastic recoil—insufficiently accounts for explaining the dynamic and methacholine-sensitive process we reported here. Imbalanced small airway pressure and/or impaired bronchiolar smooth muscle tones associated with a possible mucostasis are hypothesis to specifically address in future studies.

 Whether currently available treatment dedicated to small airways may help these patients is rather unknown. Most lines of evidence suggest a negative answer as neither inhaled corticosteroids (ICS) nor long-acting beta agonists tended to improve symptoms, because of their inability to reach the distal area of the lung [32]. On the contrary, newer formulations of ICS, such as hydrofluoroalkane propellants in solution, could have greater access to the distal airways [33, 34], but more studies are necessary to reach a conclusion, as no evidence of airway inflammation is presently shown. Weight loss and exercise training are key management cornerstones, but failure and/or relapse after intermittent therapeutic adherence are frequent. 

At a glance, we identified a different level of methacholine-induced bronchoconstriction only at distal airway level of symptomatic nonasthmatic women of a nonestablished cause.

## Figures and Tables

**Table 1:** Lung function data of the BMI-normal, overweight, and obese patients, in the entire population.

Variables	Total	BMI < 25	25 ≤ BMI < 30	BMI ≥ 30	*P* value
	*n* = 293	*n* = 182	*n* = 62	*n* = 49	
Smoking					0.2350
Yes	39 (13.31)	29 (15.93)	8 (12.90)	2 (4.08)	
No	228 (77.82)	137 (75.27)	50 (80.65)	41 (83.67)	
Former smoker	26 (8.87)	16 (8.76)	4 (6.45)	6 (12.24)	
Age (years)	41.04 (12.80)	37.91 (12.76)	45.38 (12.39)***	47.18 (9.32)**	<0.0001
BMI (kg/m^2^)	24.65 (5.41)	21.22 (2.19)	27.45 (1.51)***	33.83 (4.02)***	<0.0001
FVC (L)	3.20 (0.59)	3.36 (0.54)	2.99 (0.61)**	2.89 (0.56)***	<0.0001
FVC (% pred)	0.99 (0.14)	1.01 (0.14)	0.97 (0.13)	0.98 (0.15)	0.1094
FEV1 (L)	2.72 (0.54)	2.88 (0.52)	2.49 (0.49)**	2.42 (0.48)**	<0.0001
FEV1 (% pred)	0.98 (0.14)	0.99 (0.14)	0.94 (0.13)*	0.97 (0.16)	0.0287
FEV1/FVC (%)	0.85 (0.06)	0.86 (0.07)	0.84 (0.05)*	0.84 (0.05)*	0.0375
FVC fall (%)	11.32 (8.71)	10.66 (8.84)	10.85 (8.07)	14.37 (8.53)**	0.0111
FEV1 fall (%)	16.92 (9.96)	16.72 (10.38)	15.17 (9.40)	19.86 (8.53)*	0.0230
FVC DRS	0.03 (0.10)	0.03 (0.11)	0.02 (0.03)	0.05 (0.11)**	0.0008
FEV1 DRS	0.04 (0.13)	0.04 (0.15)	0.03 (0.05)	0.07 (0.15)**	0.0008
CI	0.68 (0.57)	0.67 (0.64)	0.72 (0.53)	0.71 (0.28)	0.1142

Data are presented as median (interquartile range) (all but smoking) and *n* (%) (smoking). BMI: body mass index; FEV1: forced expiratory volume in one second; FVC: forced volume capacity; DRS: dose-response slope; CI: closing index.

*P < 0.05 versus BMI < 25; **P < 0.01 versus BMI < 25; ***P < 0.001 versus BMI < 25.

**Table 2:** Lung function data of the BMI-normal, overweight, and obese patients, in the AHR+ population.

Variables	Total	BMI < 25	25 ≤ BMI < 30	BMI ≥ 30	*P* value
	*n* = 123	*n* = 71	*n* = 20	*n* = 32	
Smoking					0.3120
Yes	18 (14.63)	13 (18.31)	4 (20.00)	1 (3.13)	
No	93 (75.61)	51 (71.83)	14 (70.00)	28 (87.50)	
Former smoker	12 (9.76)	7 (9.86)	2 (10.00)	3 (9.38)	
Age (years)	41.50 (12.94)	37.98 (13.28)	45.57 (13.03)	46.80 (9.44)	0.0025
BMI (kg/m^2^)	25.19 (6.26)	20.66 (2.05)	27.58 (1.45)***	33.77 (4.11)***	<0.0001
FVC (L)	3.04 (0.52)	3.18 (0.45)	2.98 (0.60)	2.78 (0.54)	0.0004
FVC (% pred)	0.95 (0.13)	0.96 (0.12)	0.94 (0.11)	0.94 (0.14)	0.5261
FEV1 (L)	2.57 (0.50)	2.73 (0.47)	2.44 (0.47)**	2.31 (0.46)***	<0.0001
FEV1 (% pred)	0.93 (0.14)	0.96 (0.13)	0.90 (0.11)	0.92 (0.15)	0.1930
FEV1/FVC (%)	0.84 (0.07)	0.86 (0.04)	0.82 (0.05)	0.83 (0.05)	0.1068
FVC fall (%)	17.52 (8.42)	16.76 (9.60)	19.02 (5.15)	18.26 (7.21)	0.3434
FEV1 fall (%)	24.97 (8.15)	25.38 (9.58)	24.71 (5.29)	24.26 (6.10)	0.7683
FEV1 DRS	0.09 (0.20)	0.10 (0.22)	0.07 (0.08)	0.11 (0.18)	0.6897
FVC DRS	0.06 (0.15)	0.06 (0.18)	0.05 (0.05)	0.08 (0.13)	0.5523
CI	0.69 (0.26)	0.65 (0.25)	0.77 (0.15)	0.76 (0.24)	0.0591

Data are presented as median (interquartile range) (all but smoking) and *n* (%) (smoking). BMI: body mass index; FEV1: forced expiratory volume in one second; FVC: forced volume capacity; DRS: dose-response slope; CI: closing index.

*P < 0.05 versus BMI < 25; **P < 0.01 versus BMI < 25; ***P < 0.001 versus BMI < 25.

**Table 3:** Lung function data of the BMI-normal, overweight, and obese patients, in the AHR− population.

Variables	Total	BMI < 25	25 ≤ BMI < 30	BMI ≥ 30	*P* value
	*n* = 170	*n* = 111	*n* = 42	*n* = 17	
Smoking					0.2350
Yes	21 (12.35)	16 (14.41)	4 (9.52)	1 (5.88)	
No	135 (79.41)	86 (77.48)	36 (85.71)	13 (76.47)	
Former smoker	14 (8.24)	9 (8.11)	2 (4.76)	3 (17.64)	
Age (years)	40.70 (12.71)	37.86 (12.48)	45.29 (12.22)**	47.90 (9.32)***	0.0003
BMI (kg/m^2^)	24.25 (4.67)	21.57 (2.21)	27.39 (1.54)***	33.92 (3.98)***	<0.0001
FVC (L)	3.31 (0.62)	3.47 (0.57)	2.98 (0.62)***	3.10 (0.54)**	<0.0001
FVC (% pred)	1.02 (0.14)	1.03 (0.14)	0.98 (0.14)*	1.06 (0.13)	0.0415
FEV1 (L)	2.82 (0.55)	2.97 (0.53)	2.51 (0.50)***	2.63 (0.47)**	<0.0001
FEV1 (% pred)	1.01 (0.14)	1.02 (0.14)	0.96 (0.14)*	1.06 (0.14)	0.0226
FEV1/FVC (%)	0.85 (0.06)	0.85 (0.06)	0.84 (0.05)	0.85 (0.04)	0.5918
FVC fall (%)	6.87 (5.68)	6.81 (5.61)	6.95 (6.05)	7.05 (5.54)	0.9337
FEV1 fall (%)	11.14 (6.54)	11.26 (6.40)	10.62 (7.25)	11.58 (5.89)	0.6511
FEV1 DRS	0.006 (0.003)	0.006 (0.003)	0.005 (0.003)	0.006 (0.002)	0.4671
FVC DRS	0.003 (0.003)	0.004 (0.003)	0.003 (0.002)	0.005 (0.002)	0.7422
CI	0.67 (0.72)	0.68 (0.79)	0.70 (0.63)	0.72 (0.30)	0.5918

Data are presented as median (interquartile range) (all but smoking) and *n* (%) (smoking). BMI: body mass index; FEV1: forced expiratory volume in one second; FVC: forced volume capacity; DRS: dose-response slope; CI: closing index.

*P < 0.05 versus BMI < 25; **P < 0.01 versus BMI < 25; ***P < 0.001 versus BMI < 25.

**Table 4:** Adjusted *R* ^2^ and Spearman coefficients for the relationship between FVC DRS and FEV1 DRS.

Populations	Adjusted *R* ^2^	Spearman correlation coefficient
Entire population		
BMI < 25	0.8243	0.8999
25 ≤ BMI < 30	0.8614	0.9320
BMI ≥ 30	0.9008	0.9700
AHR+ patients		
BMI < 25	0.7988	0.8332
25 ≤ BMI < 30	0.8590	0.9030
BMI ≥ 30	0.8220	0.9284
AHR− patients		
BMI < 25	0.6698	0.6778
25 ≤ BMI < 30	0.6862	0.8032
BMI ≥ 30	0.7724	0.8728

Data shown are the adjusted *R* ^2^ calculated by the linear regression log(FEV1 DRS) = log(FVC DRS) and the Spearman correlation coefficient between the two variables log(FEV1 DRS) and log(FVC DRS), for the three levels of BMI and for our three populations (AHR+ patients, AHR− patients, and total population).

**Table 5:** Analyses of covariance for the impact of BMI on the relationship between FVC DRS (independent variable) and FEV1 DRS (predicted variable), adjusted for age and baseline respiratory values.

Parameter	Entire population			AHR+			AHR−		
	Estimate	Std. error	*P* value	Estimate	Std. error	*P* value	Estimate	Std. error	*P* value
(Intercept)	0.02	0.19	0.91	0.00	0.20	0.99	−1.74	0.52	**0.01**
Age	−0.01	0.00	0.11	0.00	0.00	0.98	−0.01	0.01	0.23
Baseline FVC	−0.13	0.18	0.46	0.19	0.24	0.44	−0.20	0.23	0.40
Baseline FEV1	0.06	0.21	0.77	0.02	0.29	0.94	0.10	0.28	0.73
Log(FVC DRS)	0.91	0.04	<**2*e ***−**16**	0.86	0.05	<**2*e ***−**16**	0.62	0.09	<**2*e ***−**16**
BMI (overweight)	−0.06	0.35	0.87	0.42	0.44	0.34	0.93	0.94	0.33
BMI (obese)	−0.57	0.30	0.06	0.11	0.33	0.74	−1.64	1.06	0.12
Log(FVC DRS) ∗ BMI (overweight)	0.00	0.07	0.96	0.18	0.12	0.15	0.17	0.16	0.30
Log(FVC DRS) ∗ BMI (obese)	−0.16	0.06	**0.01**	0.08	0.09	0.41	−0.33	0.17	**0.03**

FEV1: forced expiratory volume in one second; FVC: forced volume capacity; DRS: dose-response slope.

BMI-normal subjects were considered as the control group.

**Table 6:** Impact of BMI on the relationship between FVC DRS and FEV1 DRS—slope comparisons.

	BMI < 25	25 ≤ BMI < 30	BMI ≥ 30	*P*
Entire population	0.90 (0.04)	0.91 (0.05)	0.74 (0.04)**	0.0079
AHR+ population	0.86 (0.06)	1.04 (0.06)	0.94 (0.06)	0.4090
AHR− population	0.62 (0.09)	0.78 (0.14)	0.29 (0.06)*	0.0283

Data shown are slopes (and their standard deviations) calculated by the linear regression log(FEV1 DRS) = log(FVC DRS), adjusted for age and baseline respiratory values, for the three levels of BMI and for the three populations. The *P* value is calculated by analyses of covariance.

*P < 0.05 versus BMI < 25; **P < 0.01 versus BMI < 25.

## Abbreviation

AHR: Airway hyperresponsiveness
AHR+: Hyperreactive patients
AHR−: Nonhyperreactive patients
BMI: Body mass index
DRS: Dose-response slope
FEV1: Forced expiratory volume in one second
FVC: Forced Vital Capacity
PC20: Provocative concentration causing a 20% fall in FEV1.
